# Risk Factors of Pulmonary Arterial Hypertension and Its Relationship With Atrial Fibrillation in Patients With Obstructive Hypertrophic Cardiomyopathy

**DOI:** 10.3389/fcvm.2021.666431

**Published:** 2021-07-07

**Authors:** Changrong Nie, Changsheng Zhu, Minghu Xiao, Zhengyang Lu, Qiulan Yang, Yanhai Meng, Rong Wu, Shuiyun Wang

**Affiliations:** ^1^Department of Cardiovascular Surgery, National Center for Cardiovascular Diseases, Chinese Academy of Medical Sciences and Peking Union Medical College, Fuwai Hospital, Beijing, China; ^2^Department of Ultrasound, National Center for Cardiovascular Diseases, Chinese Academy of Medical Sciences and Peking Union Medical College, Fuwai Hospital, Beijing, China; ^3^Department of Intensive Care Unit, National Center for Cardiovascular Diseases, Chinese Academy of Medical Sciences and Peking Union Medical College, Fuwai Hospital, Beijing, China

**Keywords:** pulmonary arterial hypertension, obstructive hypertrophic cardiomyopathy, atrial fibrillation, risk facors, left ventricular outflow tract

## Abstract

**Background:** Pulmonary arterial hypertension (PH) is a common complication in patients with obstructive hypertrophic cardiomyopathy (OHCM). The risk factor of PH in patients with OHCM has not been fully elucidated, and even atrial fibrillation (AF) was considered a risk factor of PH. Thus, our study aimed to investigate risk factors of PH and the relationship between PH and AF in patients with OHCM.

**Methods:** We retrospectively enrolled 483 consecutive patients diagnosed with OHCM at Fuwai Hospital (Beijing, China) from January 2015 to December 2017. Clinical and echocardiographic parameters were compared between patients with and without PH.

**Results:** Eighty-two (17.0%) patients were diagnosed with PH in this study. Compared to patients without PH, those with PH were significantly older, had a lower body mass index (BMI), were more likely to be female and more symptomatic [New York Heart Association Class 3 or 4 symptoms], and had a higher AF prevalence. A multivariate analysis indicated that AF was an independent risk factor of PH (odds ratio [OR] 2.31, 95% confidence interval [CI] 1.03–5.20, p = 0.042). Moreover, PH was independently associated with a higher AF incidence after adjusting for age and left atrial diameter (OR 2.24, 95% CI 1.07–4.72, p = 0.034).

**Conclusion:** AF was independently associated with PH in patients with OHCM. Further, PH was significantly associated with an increased risk of AF, which suggested that AF could aggravate PH and that PH may promote AF processes, forming a vicious circle.

## Introduction

Pulmonary arterial hypertension (PH), characterized by a progressive increase in pulmonary vascular resistance, leading to right ventricular dysfunction, is a common complication in patients with hypertrophic cardiomyopathy (HCM) (1, 2). Patients with HCM and PH usually have worse outcomes, a higher incidence of atrial fibrillation (AF), and even more embolic events than patients with only HCM (3, 4). Several mechanisms, such as elevated left-side diastolic pressures, secondary to diastolic dysfunction, left ventricular outflow tract (LVOT) obstruction with mitral regurgitation, and systolic dysfunction in a minority of cases, may contribute to PH in patients with HCM (5, 6). However, risk factors of PH in patients with HCM have not been fully elucidated. PH and AF share many risk factors, and even some studies indicated that AF is a risk factor of PH in patients with HCM (5, 7, 8). Whereas, whether PH is a risk factor of AF in patients with HCM is still unknown. Therefore, this study aimed to investigate the risk factors of PH and the relationship between PH and AF in patients with obstructive HCM (OHCM).

## Materials and Methods

### Population

A total of 483 consecutive patients diagnosed with OHCM at Ward 7 of cardiovascular surgery of the Fuwai Hospital (Beijing, China) from January 2015 to December 2017 were enrolled in this study. All patients enrolled in this study were admitted to our hospital for surgical treatment, and no outpatients were included because their complete medical records cannot be obtained. We retrospectively analyzed patient echocardiographic data and divided patients into patients with PH (*N* = 82) and patients without PH (*N* = 401) groups based on the pulmonary artery systolic pressure (PASP) at initial evaluation. Clinical and echocardiographic parameters were compared between the two groups.

AF diagnosis was based on medical records and in-hospital 12-lead echocardiography, and OHCM diagnosis was based on the 2020 American Heart Association/American College of Cardiology and the 2014 European Society of cardiology guidelines, which mainly included unexplained septal hypertrophy with a thickness >15 mm or a septal cardium with a thickness >13 mm with a family history of HCM as OHCM indicators (9, 10). A LOVT gradient ≥50 mmHg at rest or during provocation was an indicator for LVOT obstruction. PH was defined as patients having a PASP >35 mmHg (11).

All study patients signed informed consent forms before enrollment, and this study was also approved by the Ethics Committee of the Fuwai hospital. All study procedures were conducted in accordance with the ethical principles stated in the Declaration of Helsinki.

### Echocardiography

Transthoracic echocardiographic studies were performed using a commercially available ultrasound system (E9 ultrasound system, GE Healthcare, Horten, Norway). Complete M-mode, two-dimensional, and Doppler studies were performed on patients in the left lateral decubitus or supine position, using standard parasternal, apical, and subcostal approaches. Diameters of cardiac chambers were presented as the maximum value of the anteroposterior diameter in cardiac cycles. Maximum left ventricular (LV) wall thickness was the greatest dimension measured at any site within the LV chamber at end diastole. The LVOT gradient was scanned with a continuous-wave Doppler ultrasound to measure the maximal outflow velocity and was estimated using the simplified Bernoulli equation. PASP was estimated from the tricuspid regurgitant jet velocity using the modified Bernoulli equation and adding to right atrial pressure (RAP) when patients' tricuspid regurgitation jet can be detected. Moreover, we measured mean pulmonary arterial pressure (PAP) through peak pulmonary regurgitation (PR) velocity among those who failed to measure PASP through the tricuspid regurgitation jet. Furthermore, mean PAP was calculated using the following formula: mean PAP = 4(peak PR velocity)^2^ + RAP. Patients with a mean PAP >25 mmHg were also considered to have PH. When a patient was suspected of having PH, we would detect PR end-velocity to estimated pulmonary aterial diastolic pressure (PADP) and then use an equation of mean PAP = 2/3rd of PADP + 1/3rd of PASP to calculate PASP. In this study, 79 patients diagnosed with PH through TR jet velocity and 3 patients through peak PR velocity. The RAP (5, 10, and 15 mmHg) was estimated in the subcostal view based on the inferior vena cava size and collapsibility during inspiration at rest (12, 13). More details can be found in our previous publication (14).

### Statistical Analysis

**Figure 1:**
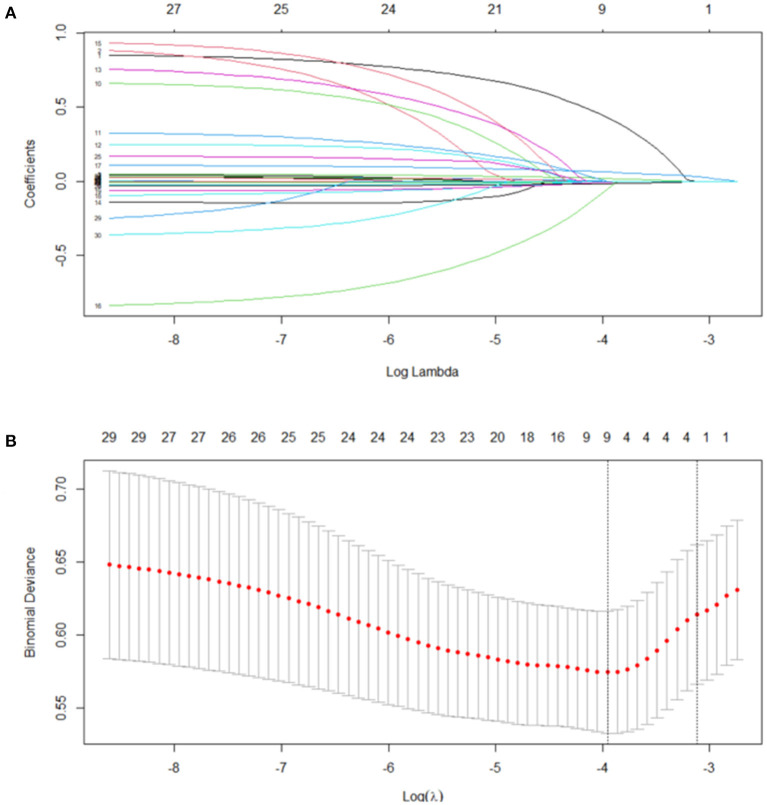
Feature selection using the least absolute shrinkage and selection operator binary logistic regression model. **(A)** Binomial deviance was plotted against the log (lambda) sequence. **(B)** A coefficient profile plot was produced against the log (λ) sequence. In this study, predictors were selected according to the minimum criteria. Four nonzero coefficients (age, left atrial diameter, Pulmonary arterial hypertension and rest left ventricular outflow gradient) were selected to construct a Atrial fibrillation risk predictive model.

Results are presented as mean ± standard deviations or as number (percentage), as appropriate. Independent variables were compared using Student's *t*-test, and continuous variables were compared using the Mann-Whitney U test continuous variables; the χ2 test or Fisher's exact test was used to compare nominal variables, as appropriate. A least absolute shrinkage and selection operator (LASSO) logistic regression analysis was performed to select variables associated with AF to be included in a multivariate logistic regression model (Figure 1). All *p*-values were two-tailed, and a *p*-value < 0.05 was considered statistically significant. SPSS version 26.0 (IBM), R version 3.6.0, and GraphPad Prism version 8.0 (GraphPad Software Inc., La Jolla, CA, USA) were used for calculation and illustration, respectively.

## Results

### Baseline Patient Characteristics

A total of 82 (17.0%) patients were diagnosed with PH at initial echocardiographic evaluation. The clinical characteristics of patients with and without PH are presented in Table 1. Compared to patients without PH, those with PH were significantly older (50.9 ± 13.0 vs. 44.6 ± 14.8, *p* < 0.001), had a lower body mass index (BMI) (24.3 ± 3.8 vs. 25.3 ± 3.8, *p* = 0.031), were more likely to be female [50 (61.0%) vs. 146 (36.4%), *p* < 0.001] and more symptomatic [New York Heart Association Class 3 or 4 symptoms] [73 (89.0%) vs. 316 (78.8%), *p* = 0.048], and had a higher AF prevalence [16 (19.5 %) vs. 30 (7.5%), *p* = 0.001]. Moreover, values of echocardiographic parameters such as left atrial diameter, LVOT gradient, and the proportion of moderate or severe mitral and tricuspid regurgitations were significantly higher in patients with PH than those without PH.

**Table 1:** Baseline characteristics of the study population.

**Variables**	**OHCM with PH (*N* = 82)**	**OHCM without PH (*N* = 401)**	***P***
Female (*N*, %)	50 (61.0)	146 (36.4)	<0.001
Age (y)	50.9 ± 13.0	44.6 ± 14.8	<0.001
BMI (kg/m^2^)	24.3 ± 3.8	25.3 ± 3.8	0.031
Heart rate (beats/min)	71.0 ± 9.3	71.8 ± 8.7	0.421
Systolic blood pressure (mmHg)	120.1 ± 15.72	121.4 ± 14.3	0.464
Diastolic blood pressure (mmHg)	71.4 ± 9.4	72.2 ± 9.94	0.490
Smoking (*N*, %)	22 (26.8)	157 (39.2)	0.048
NYHA class III or IV (*N*, %)	73 (89.0)	316 (78.8)	0.048
**Concomitant disease**			
Atrial fibrillation (*N*, %)	16 (19.5)	30 (7.5)	0.001
Hyperlipemia (*N*, %)	17 (20.7)	87 (21.7)	0.963
Diabetes (*N*, %)	2 (2.4)	13 (3.2)	0.974
Hypertension (*N*, %)	20 (24.4)	104 (25.9)	0.878
**Clinic presentation**			
Chest pain (*N*, %)	35 (42.7)	152 (37.9)	0.493
Amaurosis (*N*, %)	14 (17.1)	102 (25.4)	0.141
Palpitation (*N*, %)	18 (22.0)	93 (23.2)	0.921
Syncope (*N*, %)	27 (32.9)	104 (25.9)	0.246
**Echocardiographic indices**			
Left atrial diameter (mm)	48.7 ± 7.6	45.2 ± 7.0	<0.001
LVEDD (mm)	41.9 ± 7.1	42.5 ± 4.9	0.322
RVEDD (mm)	20.8 ± 2.0	20.9 ± 2.6	0.833
IVST (mm)	17.7 ± 3.9	18.6 ± 4.3	0.084
Rest LVOT gradient (mmHg)	78.1 ± 33.8	66.8 ± 34.0	0.006
PASP (mmHg)	43 ± 7.9	28.7 ± 3.7 (*N* = 102)	<0.001
LVEF (%)	70.1 ± 6.3	71.1 ± 5.5	0.173
Moderate or severe MR (*N*, %)	57 (69.5)	198 (49.4)	<0.001
Moderate or severe TR (*N*, %)	47 (57.3)	39 (9.7)	<0.001
**Medical therapy**			
β receptor blocker (*N*, %)	76 (92.7)	356 (88.8)	0.395
Calcium channel blocker (*N*, %)	26 (31.7)	115 (28.7)	0.677

*OHCM, obstructive hypertrophic cardiomyopathy; PH, pulmonary arterial hypertension; BMI, body mass index; NYHA, New York Heart Association; HCM, hypertrophic cardiomyopathy; LVEDD, left ventricular end-diastolic diameter; RVEDD, right ventricular end-diastolic diameter; IVST, interventricular septal thickness; LVOT, left ventricular outflow tract; LVEF, left ventricular ejection fraction; MR, mitral regurgitation; TR, Tricuspid regurgitation*.

### PASP and Clinical Parameters

**Figure 2:**
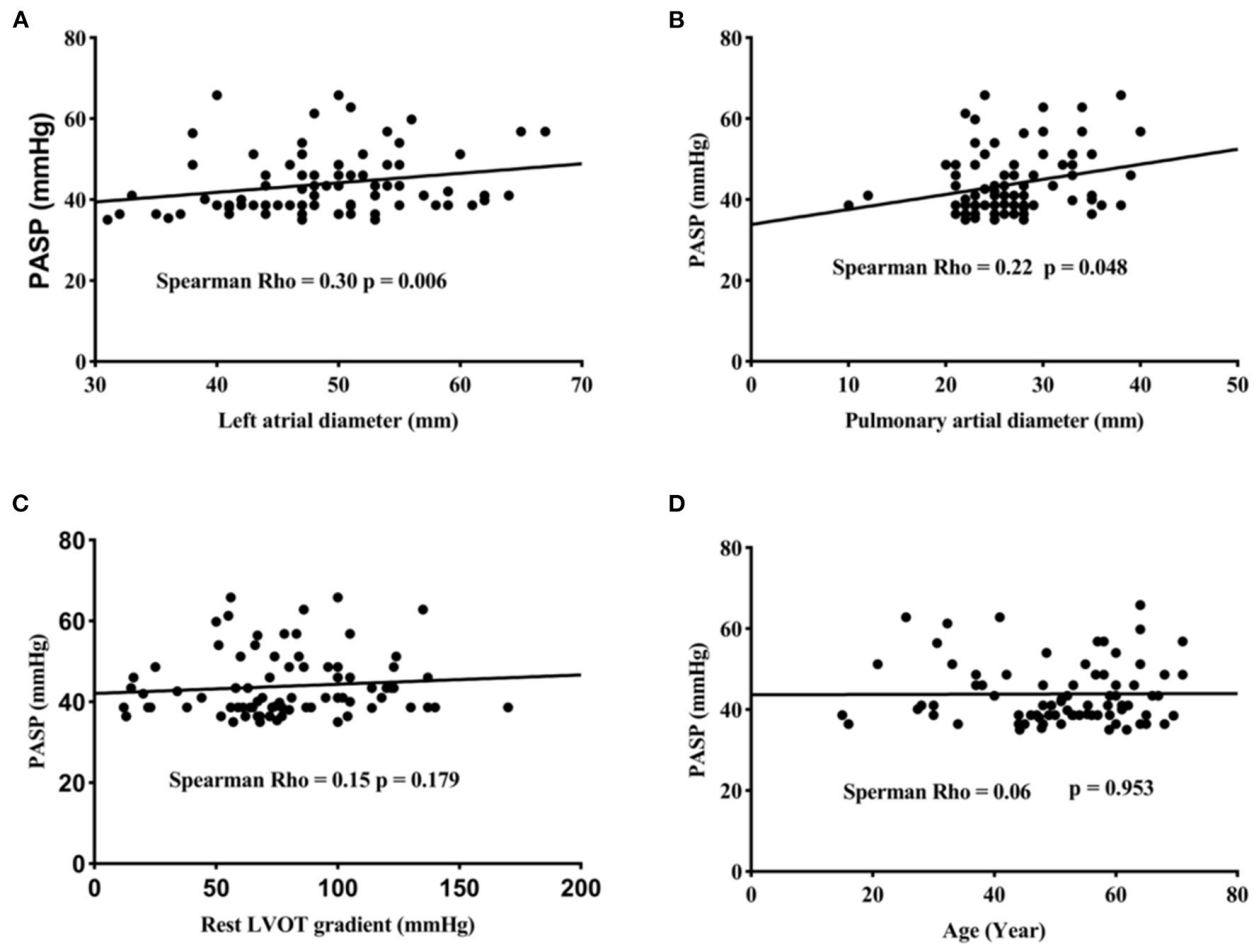
PASP increased with left atrial diameter **(A)** and Pulmonary arterial diameter **(B)**. No relationship was found between PASP and the rest left ventricular outflow tract gradient **(C)**. Pulmonary artery systolic pressure (PASP) is not correlated with age **(D)**.

**Figure 3:**
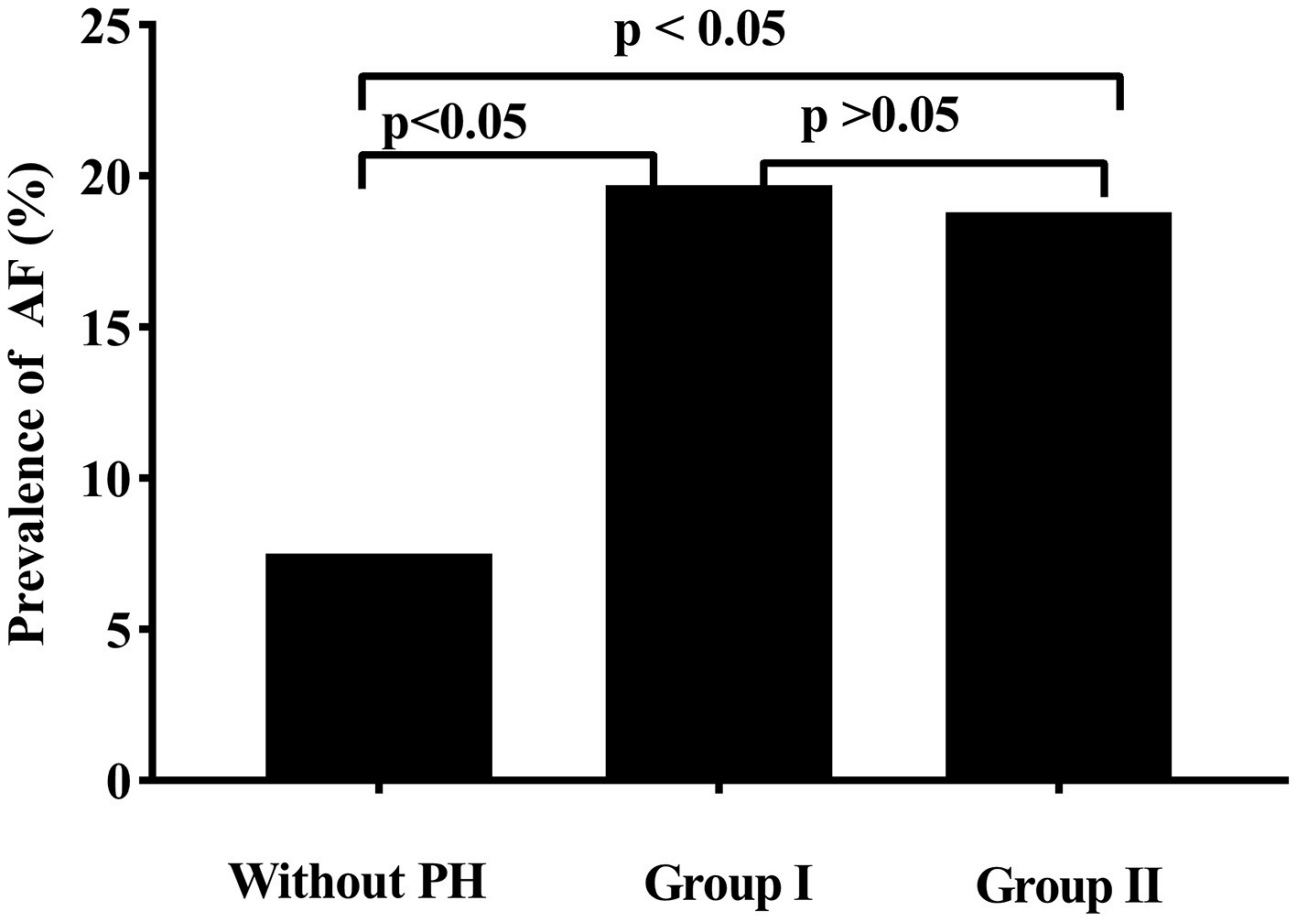
Compared to patients without pulmonary arterial hypertension (PH), patients with PH had a significantly atrial fibrillation (AF) higher incidence. No diffidence in AF prevalence was found between patients with a pulmonary artery systolic pressure (PASP) <50 mmHg (Group I) and patients with a PASP ≥ 50 mmHg (Group II).

Relationships between age, left atrial diameter, right ventricular diameter, LVOT gradient and PASP are presented in Figure 2. In this study, PASP reportedly increased when left atrial diameter (Spearman rho = 0.30, *p* = 0.006) and pulmonary aterial diameter (Spearman rho = 0.22, *p* = 0.048) increased. However, we did not find a direct association between PASP and the rest LVOT gradient (Spearman rho = 0.15, *p* = 0.179) and age (Spearman rho = 0.06, *p* = 0.589). When we divided patients with PH into two groups based on PASP (PASP ≤ 50 mmHg, group I; and >50 mmHg, group II), AF prevalence was higher in patients with PH than in patients without PH; however, AF prevalence was the same in group I and group II (Figure 3).

### Analysis of Risk Factors

Risk factors associated with PH and AF selected from the LASSO logistic regression analysis are present in Tables 2, 3. In the multivariate analysis, in addition to female sex, BMI, left atrial diameter, and moderate or severe mitral and moderate or severe tricuspid regurgitation, AF was found to be an independent risk factor of PH (odds ratio [OR] 2.31, 95% confidence interval [CI] 1.03–5.20, *p* = 0.042). Furthermore, the multivariate analysis revealed that, in addition to age, left atrial diameter, and rest LVOT gradient, PH (OR 2.24, 95% CI 1.07–4.72, *p* = 0.034) was an independent risk factor of AF (Univariate analysis of the differences between patients with and without AF was presented in Supplementary Table 1).

**Table 2:** Logistic regression models for analysis of the risk factors of PH.

**Variables**	**Univariable (OR, 95% CI)**	***P***	**Multivariable (OR, 95% CI)**	***P***
Age	1.03 (1.02–1.05)	<0.001		
Female	2.73 (1.68–4.45)	<0.001	2.73 (1.51–4.93)	0.001
BMI	0.93 (0.88–0.99)	0.032	0.90 (0.83–0.98)	0.012
NYHA class III or IV	2.18 (1.05–4.54)	0.037		
Atrial fibrillation	3.00 (1.55–5.81)	0.001	2.31 (1.03–5.20)	0.042
LAD	1.07 (1.03–1.11)	<0.001	1.08 (1.03–1.13)	0.002
Resting LVOT gradient	1.01 (1.003–1.02)	0.007		
Moderate or severe MR	2.34 (1.41–3.89)	0.001		
Moderate or severe MR	12.5 (7.20–21.57)	<0.001	9.47 (5.29–16.94)	<0.001

*OR, odds Ratio; LAD, left atrial diameter; other abbreviations are as in Table 1*.

**Table 3:** Logistic regression models for analysis of the risk factors of AF.

**Variables**	**Univariable (OR, 95% CI)**	***P***	**Multivariable (OR, 95% CI)**	***P***
Age	1.05 (1.02–1.07)	0.001	1.04 (1.01–1.07)	0.006
PH	3.00 (1.55–5.81)	0.001	2.24 (1.07–4.72)	0.034
LAD	1.11 (1.06–1.16)	<0.001	1.10 (1.05–1.15)	<0.001
Rest LVOT gradient	0.988 (0.978–0.998)	0.014	0.98 (0.97–0.99)	<0.001

*Abbreviations are as in Table 1 or Table 2*.

## Discussion

The main findings of this study are that, first, PH prevalence in this study population is ~17.0%. Second, in addition to female sex, BMI, left atrial diameter, moderate or severe mitral and tricuspid regurgitation, AF was found to be an independent risk factor of PH. Third, PH was independently associated with a higher incidence of AF, even though there was no relationship between PASP and AF prevalence.

### PH Prevalence in HCM Patients

Studies concerning PH prevalence in patients with HCM were limited. PH prevalence in this single-center cohort study was 17.0%, and PH frequency was similar to that noted in Musumeci's study, which had a PH incidence of 11.4% at initial evaluation (3). In a highly selected population of patients with OHCM undergoing septal reduction therapy, PH prevalence was 53% (15). In another large HCM population, the incidence of PH was 38.2%, which included 37.5% of patients with OHCM (4). This remarkable difference in PH prevalence between studies may be caused by different PH thresholds or different PH measurement methods in the literature. Moreover, selection bias, ethnic differences, and geographical differences may also significantly contribute to this difference.

### Risk Factors of PH Patients

There were several factors that may contribute to the development of PH in patients with HCM. Consistent with previous studies, our findings confirmed that PH has a strong female predominance, which suggested that sex-related differences in the production and metabolism of hormones may affect the pulmonary vasculature (4, 11, 15, 16). Further, it is previously known that left-side heart disease increases left atrial pressure, which passively reaches the pulmonary vasculature and consequently leads to vascular resistance (6). This can explain our findings that left atrial diameter and mitral regurgitation were associated with a PH higher incidence. Moreover, increased left atrial pressure led to atrial stretch and remolding, resulting in an increase in repolarization dispersion; this may allow ectopic triggers to maintain AF (7). Moreover, atrial dysfunction caused by AF usually elevates left atrial pressure, which leads to the development of post-capillary pulmonary hypertension and, subsequently, aggravated PH (5, 8). Moreover, higher BMI was found to be independently associated with a lower incidence of PH in our present study. Several studies have demonstrated a negative correlation between BMI and pulmonary arterial pressure previously, and even excess BMI was found to be a mortality protective factor in patients with PH (17–19). The obesity paradox was common in patients with cardiovascular disease, even though the mechanisms of this paradox have not been fully elucidated yet. A potential mechanism of the obesity paradox in patients with PH was that an excessive expression of sympathetic nervous activity differentially regulated the pulmonary vascular tone, thereby limiting unfavorable effects of severe PH (20). Surprisingly, although patients with PH were older than those without PH, we did not find any association between age and PH after adjusting for other variables. Furthermore, no relation was found between age and PASP in our study population. Many investigators believed that age-related ventricular dysfunction may contribute to a higher PH incidence in HCM patients (15). However, morphological features and genetic variants of HCM differed among different age groups; this had a great impact on ventricular function in patients with HCM (21, 22). Based on existing studies, the relationship between age and PH in patients with HCM remains unsolved. Further studies are needed to focus on PH mechanisms in patients with OHCM.

### Impact of PH on AF

Previous studies reported a higher incidence of supraventricular arrhythmia, AF, and stroke events in patients with PH than those without PH (3, 23, 24). However, the impact of PH on AF has not been fully illustrated. In this study, we confirmed that PH was independently associated with an increased AF risk. The potential mechanism between PH and AF may be as follows. First, PH elevates right atrial pressure, leading to right atrium dilation and stretching; this results in electrical remolding, which increases propensity for AF (25). Some changes, such as the prolongation of sinus restoration time, atrial conduction slowing with a significant decrease in conduction velocity, and an increase in activation times at a global and regional level, were observed in the right atrium of patients with PH (26). An animal experiment demonstrated that PH promoted right ventricular hypertrophy due to pressure overload, resulting in a right pressure increase (27). As a result of right atrium mechanical loading, tissue fibrosis and electrical abnormalities, including altered conduction and repolarization properties, were observed (27). Moreover, an increased expression of genes related to hypertrophy, inflammation, apoptosis, and fibrosis were observed in the PH group compared to controls (27). These genes and abnormalities result in a proarrhythmic substrate that increases AF vulnerability. Thus, further studies in the future need to be conducted with an emphasis on the mechanism between right-side heart dysfunction and AF.

### Clinical Implication

Pulmonary hypertension is a common complication in patients with HCM and is usually associated with worse survival. A large population study reported a 40% risk of death at 10 years after the initial evaluation of OHCM in patients with PH who were treated medically; this rate is two-fold higher than in OHCM patients without PH (4). Moreover, higher incidences of AF and strokes were observed in patients with PH than in those without PH (3). Conversely, the risk of death at 10 years postoperatively in OHCM patients with PH was similar to those without PH, meaning that relieving the outflow gradient abolished unfavorable outcomes associated with PH in patients with OHCM (4). Another study confirmed that surgical myectomy treatments could reduce right ventricular systolic pressure in HCM patients with PH, especially in patients with moderate or severe PH (15). Besides, PH continued to decline during follow-up among those patients (15). Relieving the outflow gradient in patients with OHCM could decrease PH via improving left ventricular function and reducing left atrial loading. Therefore, we expect that early and timely diagnosis and appropriate treatments will reduce PH in OHCM patients and will eventually improve patient symptoms and long-term prognosis.

### Limitation

Our study was a single-center, retrospective, observational study with some inherent limitations. Due to the relatively small sample size of patients diagnosed with PH, our study findings should be prudently extrapolated to other centers, and further studies with a larger population are needed to confirm our results. Moreover, because of the retrospective nature of data collection, some factors, such as right atrial diameter, main pulmonary arterial diameter, level of right atrial fibrosis, and the electric remolding of the right atrium could not be evaluated in this study. This limited our ability to illustrate the role of PH in AF development directly. Moreover, PH assessment was based on echocardiography, not right heart catheterization, which is the gold standard for evaluating pulmonary pressure.

In conclusion, in addition to female sex, BMI, left atrial diameter, moderate or severe mitral and tricuspid regurgitation, AF was found to be independently associated with PH in patients with OHCM. Further, PH was independently associated with an increased risk of AF, which suggested that AF could aggravate PH and that PH may promote AF processes, forming a vicious circle.

## Data Availability Statement

The original contributions presented in the study are included in the article/Supplementary Material, further inquiries can be directed to the corresponding author.

## Ethics Statement

The studies involving human participants were reviewed and approved by Ethics Committee of Fuwai Hospital. The patients/participants provided their written informed consent to participate in this study.

## Author Contributions

CN and SYW contributed to the design of the study. CN, CZ, and MX contributed to the data collection and analysis, while all authors contributed to data interpretation. CN drafted the manuscript and ZL, QY, YM, and RW contributed significantly to the preparation. All authors critically revised the manuscript and gave final approval and agreed to be accountable for all aspects of the work, to ensure its integrity and accuracy.

## Conflict of Interest

The authors declare that the research was conducted in the absence of any commercial or financial relationships that could be construed as a potential conflict of interest.
