# Supplementary material for: Risk Factors of Pulmonary Arterial Hypertension and Its Relationship With Atrial Fibrillation in Patients With Obstructive Hypertrophic Cardiomyopathy
Source: Front Cardiovasc Med. 2021 Jul 7;8:666431. doi: 10.3389/fcvm.2021.666431 (PMC8292618; doi:10.3389/fcvm.2021.666431)
Supplement: Supplementary file 1 [file Table_1.DOCX]

| Supplemental table 1 Patient baseline characteristics stratified by patients with and without AF | | | |
| --- | --- | --- | --- |
| Variables | Patients with AF  (N=46) | Patients without AF  (N=437) | P-values |
| Female,N(%) | 20 (43.5) | 176 (40.3) | 0.793 |
| Age (y) | 52.9±12.7 | 44.9±14.64 | <0.001 |
| Heart rate (beats/min) | 73.5±14.2 | 71.5±8.0 | 0.135 |
| Systolic blood pressure (mmHg) | 119.7±15.2) | 121.4±14.5 | 0.476 |
| Diastolic blood pressure (mmHg) | 72.2±8.4 | 72.1±10.0 | 0.957 |
| BMI（(kg/m^2^） | 26.2±4.5) | 25.0±3.7 | 0.05 |

| smoking = 1 (%) | 16 (34.8) | 163 (37.3) | 0.86 |
| --- | --- | --- | --- |
| NYHA = 1 (%) | 34 (73.9) | 355 (81.2) | 0.319 |

| **Concomitant disease** | |  |  |  |
| --- | --- | --- | --- | --- |
| Diabetes (N, %) | | 3 (6.5) | 12 (2.7) | 0.338 |
| Hyperlipemia (N, %) | | 15 (32.6) | 89 (20.4) | 0.083 |
| Hypertension (N, %) | | 17 (37.0) | 107 (24.5) | 0.096 |
| \| PH (N,%) \| 16 (34.8) \| 66 (15.1) \| 0.001 \| \| --- \| --- \| --- \| --- \| | | 16 (34.8) | 66 (15.1) | 0.001 |
| **Echocardiographic indices** |  | |  |  |
| Left atrial diameter (mm) | 50.7±6.3 | | 45.3±7.1 | <0.001 |
| IVST (mm) | 17.6±3.6 | | 18.5±4.3 | 0.15 |
| LVEDD (mm) | 41.9±6.9 | | 42.4±5.1 | 0.51 |
| LVEF (mean (SD)) | 70.7±4.8 | | 70.9±5.8 | 0.812 |
| LVPWT (mean (SD)) | 12.0±2.6 | | 11.8±2.5 | 0.579 |
| RVEDD (mm) | 21.3±2.7 | | 20.8±2.5 | 0.203 |
| Moderate or severe MR (N, %) | 22 (47.8) | | 233 (53.3) | 0.579 |
| Moderate or severe TR (N, %) | 14 (30.4) | | 72 (16.5) | 0.031 |
| Rest LVOT gradient (mm Hg) | 56.9±28.8 | | 69.9±34.5 | 0.013 |
| **Medical therapy** |  | |  |  |
| β receptor blocker (N, %) | 44 (95.7) | | 388 (88.8) | 0.234 |
| Calcium channel blocker (N, %) | 13 (28.3) | | 128 (29.3) | 1 |

OHCM, obstructive hypertrophic cardiomyopathy; PH, pulmonary arterial hypertension; BMI, body mass index; NYHA, New York Heart Association; HCM, hypertrophic cardiomyopathy; LVEDD, left ventricular end-diastolic diameter; RVEDD, right ventricular end-diastolic diameter; IVST, interventricular septal thickness; LVOT, left ventricular outflow tract; LVEF, left ventricular ejection frac tion; MR, mitral regurgitation; TR, Tricuspid regurgitation; AF, atrial fibrillation.
